# Efficacy of Lidocaine Topical Solution in Reducing Discomfort Reaction of Horses to Intramuscular Vaccination

**DOI:** 10.3390/ani12131659

**Published:** 2022-06-28

**Authors:** Catherine Torcivia, Sue McDonnell

**Affiliations:** 1 Department of Clinical Studies, New Bolton Center, University of Pennsylvania School of Veterinary Medicine, Kennett Square, PA 19348, USA; torcivia@upenn.edu; 2 Havemeyer Equine Behavior Lab, Section of Reproduction and Behavior, Department of Clinical Studies, New Bolton Center, University of Pennsylvania School of Veterinary Medicine, Kennett Square, PA 19348, USA

**Keywords:** horse, injection aversion, needle shy, topical anesthesia, welfare, low-stress veterinary care

## Abstract

**Simple Summary:**

Intramuscular injection for vaccination and venipuncture for blood sampling are necessary for routine preventative health care for horses. A considerable proportion of horses react to these procedures, particularly as a needle pierces the skin. Many progress to become “needle shy.” Avoidance behaviors often quickly escalate to dangerous levels. Our clinical experience suggests that topical numbing solutions shown to reduce needle discomfort in other species also help horses better tolerate needles. To critically evaluate this clinical impression, 78 ponies were divided into three groups, each with either 5% or 10% lidocaine solution (commercially available preparations) or 0% control (sterile water) applied two minutes before each of two intramuscular vaccinations. Personnel handling the ponies, performing injections, scoring behavior reactions and analyzing data were unaware of treatment assignments. For both lidocaine treatments, behavioral reactions were lower than control treatment and not significantly different from one another. Fewer than 15% of lidocaine-treated ponies (7 of 51) had greater than a slight flinch reaction, compared to more than half (55%) of control group ponies (15 of 27). This clearly demonstrates that topical anesthetic can effectively reduce the behavior reaction of horses to intramuscular injection, providing convincing support for its routine use to improve animal welfare and care staff safety.

**Abstract:**

Vaccinations via intramuscular injection are a key component of preventative health care in horses. Development of problematic behavioral aversion to injections is quite common. Our clinical impression has been that topical anesthetic applied to injection sites can reduce the behavioral reaction; however, this has not been critically tested. To blindly evaluate efficacy, either 5% or 10% topical lidocaine solution or 0% control was applied to injection sites for 78 ponies two minutes before intramuscular vaccination. Mean reaction scores on a scale of 0–3 were 0.84 (se 0.18) for 5% lidocaine solution, 0.62 (se 0.14) for 10% lidocaine solution, compared to 1.30 (se 0.19) for 0% control solution. Reaction scores for both the 5% and 10% lidocaine were significantly lower than for the control group. Additionally, the proportion of subjects with a reaction greater than a slight flinch was 2 of 25 for the 5% lidocaine, 5 of 26 for the 10% lidocaine and 15 of 27 for the 0% lidocaine control. For both the 5% and 10% lidocaine groups, the proportion differed significantly from the control. The difference between the 5% and 10% lidocaine groups was not statistically significant. These findings confirm our clinical impression that application of topical anesthetic just two minutes in advance of intramuscular injection can effectively reduce the behavior reaction of horses.

## 1. Introduction

Administration of vaccinations via intramuscular injection is a key component of preventative health care in horses. However, injection aversion, commonly referred to as needle shyness, is a common equine behavior problem that may lead to difficulty vaccinating horses and often non-compliance with recommended vaccination protocols. A recent survey study of equine veterinarians in the UK reported that 92% of respondents indicated that they encountered injection-shy horses at least a few times each month, with 45% indicating at least a few times each week [1]. In addition to affecting the quality of veterinary care that horses will safely tolerate, injection shyness poses a danger to handlers and veterinarians if horses display animated avoidance responses. Finding ways to increase comfort and compliance with injections in horses is therefore of interest. The use of a topical anesthetic may provide a relatively simple means to accomplish this.

In children, topical lidocaine [2] or combination lidocaine–prilocaine preparations [3,4,5] applied before injection has been shown to reduce pain responses for intramuscular vaccination [2,3,5] as well as for caudal epidural anesthesia infusion [4]. Similarly, in dogs, topical lidocaine–prilocaine applied 60 min before intravenous catheter placement reduced behavioral reactions [6]. Cats that were treated with topical lidocaine–prilocaine 60 min in advance of catheter placement tended to struggle less compared to controls, such that catheters were successfully placed without sedation in 60% of the treated cats compared to 38% of controls [7]. In another study, only one of twelve sedated cats that had lidocaine–prilocaine cream applied 20 min in advance of cephalic intravenous catheter placement had a behavioral reaction to catheter placement, compared to 11 of the 12 sedated control cats [8].

There are limited data regarding the effectiveness of topical anesthetics in horses. Topical anesthetic creams (lidocaine–prilocaine and lidocaine–tetracaine) have been shown to increase the mechanical nociceptive threshold (pressure to evoke withdrawal reflex in response to 3 mm-diameter blunt-tipped instrument) at perineural injection sites in the distal limb [9]. For episioplasty in mares, topical lidocaine–prilocaine applied to the labia was found to be as effective at producing anesthesia for the procedure as local infiltration of injectable lidocaine into the labia [10]. In addition, topical anesthetic eliminated the need for twitching of mares during application of the anesthetic as well as deformation of the labia caused by lidocaine infiltration.

The authors have used and recommended topical lidocaine creams and solutions for injections or venipuncture for horses, particularly within the context of behavior modification rehabilitation for injection aversion [11,12,13]. Our clinical experience suggests that for most small-volume aqueous intramuscular treatments or for intravenous needle sticks, it is the piercing of the skin that elicits the principal discomfort reaction. And our assumption as well as clinical impression has been that topical lidocaine can decrease a horse’s discomfort reaction. To date, however, we are unaware of any systematic evaluation of efficacy of topical numbing agents for this purpose in horses. Therefore, the aim of our study was to blindly evaluate effectiveness of application of topical lidocaine in reducing a discomfort reaction to intramuscular injection in horses. Additionally, most topical anesthetics, and all that have been used in previously published work mentioned above, recommend application 60 min or more before a procedure. In equine practice, a one-hour or greater latency to effect may represent a potential limitation for widespread routine use. Our current study employed two commercially available topical lidocaine solutions, one 5% lidocaine and the other 10% lidocaine, both purported to produce skin anesthesia in humans within two minutes of application. Our hypothesis was that the application of either 5% or 10% topical lidocaine solution to vaccination sites two minutes in advance would result in an observably lower behavioral reaction to intramuscular injection compared to the control (0% solution) treatment. We further wanted to compare the effectiveness of the two commercially available formulations with differing concentrations of lidocaine. 

## 2. Materials and Methods

### 2.1. Subjects

The subjects included 78 Shetland-type ponies, ranging in age from 9 months to 22 years. Seventy-two of these (thirty-five intact males and thirty-seven females) were lifelong residents of a herd of semi-feral Shetland-type ponies maintained at the University of Pennsylvania School of Veterinary Medicine’s New Bolton Center. This herd was established in 1994 as a model for the study of reproductive physiology and behavior of horses living continuously under natural social and environmental conditions. Handling is mostly limited to periodic routine health care. The remaining 6 subjects (1 intact male and 5 recently castrated males) had been removed from the herd within the previous 6 months for transition to domestic handling and management as teaching animals at the same University farm. The 78 animals were divided into three groups, systematically balanced for age and sex. The 6 ponies that had been removed from the semi-feral herd were equally divided among the three groups. Each group was then randomly assigned to one of three different treatments: 0% lidocaine (control; sterile water), 5% lidocaine solution (Uber Numb Spray^®^, UberScientific, Lexington, KY, USA) or 10% lidocaine solution with epinephrine (PAINFREE Numbing Spray, FZCO Worldwide, Denmark). 

### 2.2. Study Site and General Handling Procedure

The study was carried out within the context of annual spring routine herd health care procedures, conducted over the course of two weeks in late February and early March of 2022. The battery of health care procedures for all subjects included measure of height and weight using weight estimation girth tape, body condition scoring, palpation and size estimation of testicles, administration of oral anthelmintic and administration of two intramuscular vaccinations. A 1 mL 5-way vaccine for Eastern equine encephalomyelitis, Western equine encephalomyelitis, tetanus, Equine influenza and rhinopneumanitis (Vetera^®^ Beohringer Ingelheim, St. Joseph, MO, USA; or Prestige 5^®^ Merck Animal Health, Omaha, NE, USA) was administered into the left neck and a 2 mL rabies vaccine (Imrab Large Animal^®^, Merial Inc., Athens, GA, USA) into the right neck. 

**Figure 1:**
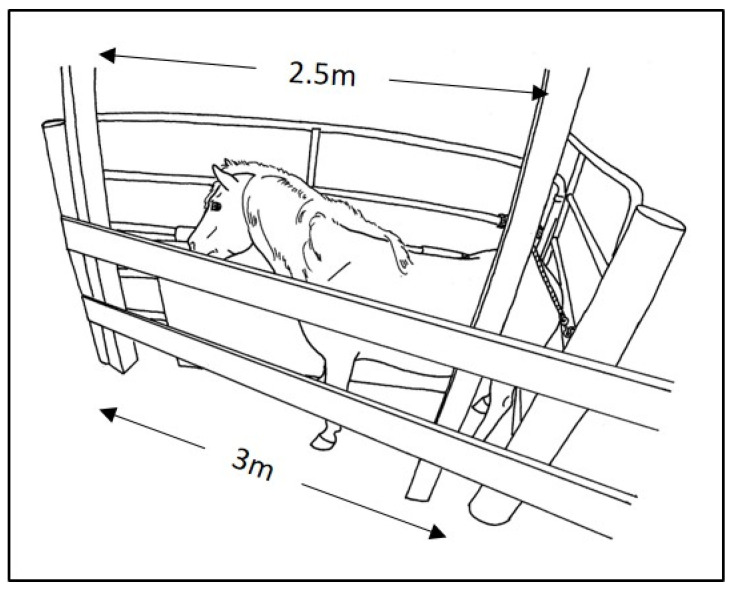
Temporary holding stall for battery of health care procedures.

All handling and procedures were performed by two equine behaviorists (CT, SM), each with extensive experience performing the various equine health care procedures using low-stress equine handling methods [11]. Both were also experienced in working with these particular animals. For the 72 semi-feral herd ponies, the procedures were conducted within their pasture environment. As is customary when working with this herd, harem or bachelor bands, one at a time, were first separated from the remainder of the herd as they passed through a familiar gated laneway. For the battery of procedures, each pony within a band was then individually enclosed in a triangular holding stall depicted in Figure 1. An aluminum 5-rail livestock gate, approximately 1.5 m high, was hinged to posts at each end of a 3 m stretch of 2-board wooden fence. One of these gates was secured at a right angle to the wooden fence, forming a corner to serve as the back stop for the stall. This gate spanned the 5 m laneway, forming a right angle with fencing on the opposite side of the laneway. The 2.5 m gate (with 5 cm-thick vinyl-covered foam sports padding) on the other end of the wooden fence was hinged to swing freely. To load a subject, the hinged 2.5 m gate was swung wide open, while the subject pony was enticed with grain and/or guided along the fixed gate into the corner and along the wooden fence. As the pony approached along the wooden fence, the 2.5 m hinged gate was closed back around the pony toward the corner. The hinged gate was then secured with a soft cotton 3 c-diameter lead rope behind the pony. The length of the rope was adjusted to custom-fit the enclosure width to the various sized ponies. The goal was to offer enough space to allow for ease of forward and backward as well as lateral movement, but not enough for turning all the way around. The open-rail arrangement enabled the subject pony to maintain visual and tactile contact with nearby family members, which often continued to interact with the subject through the rails of the gates. All subjects had had previous experience with this protocol for family separation from the remainder of the herd and then for individual enclosure within the holding stall. 

Procedures were performed by reaching over or between the rails of the stall, enabling protected contact for the handlers and to some extent for the animal. For 8 of the 72 herd ponies, a halter or lead rope collar was also applied, primarily to be able to guide the head and neck above the top wooden rail to comfortably access the mouth for oral deworming and/or the injection sites on the neck. As is performed routinely with this herd, scratching at the withers or food enticement, distraction or positive reinforcement for compliance were also used “to effect” to improve comfort with the confinement and/or procedures (Purina Equine Senior^®^, Land O’Lakes, Inc., Gray Summit, MO, USA). For the 6 former herd animals, the procedures were conducted in a similarly sized rectangular pony examination stall within their domestic housing unit. All procedures were video-recorded using a GoPro Hero 4 camera (GoPro, San Mateo, CA, USA).

### 2.3. Topical Treatment Application and Vaccine Administration

To enable handlers to remain blind to treatments, the topical solutions were pre-measured and loaded into 1 mL slip tip syringes (BD Slip-tip Tuberculin, Becton Dickenson and Company, Franklin Lakes, NJ, USA) with coded labels. Both commercial lidocaine solution products are packaged as sprays; however, because these ponies were naïve to spray application, we chose to apply the liquid directly to the skin. Since this work was carried out when ponies’ winter coats were long and thick, to mark each site for application of topical treatment and subsequent injection, an approximately 2 × 2 cm patch of hair was trimmed to about 5 mm in length using iris scissors. A 0.5 mL volume of topical treatment was applied to the trimmed patch on each side of the neck and rubbed onto the skin using a gloved finger. Two minutes after application of the topical treatment, vaccines were administered using 3 mL syringes with 22-gauge (G) 1-inch hypodermic needles (BD Plastipak Luer-Lok Tip with Precision Glide Needle, Becton Dickenson and Company, Franklin Lakes, NJ, USA). The “order of go” for bands as well as for individuals within bands was a matter of convenience. As it turned out, the three treatments were fairly evenly distributed among bands, and similarly, the order of treatments reasonably well-distributed among individuals within bands.

### 2.4. Behavior Scoring

Approximately one month following completion of the animal work, the video recordings were reviewed and scored independently by each of the same two handlers who had conducted the procedures. Again, this was carried out without knowledge of the treatment group assignments. For each of the two injections (left and right neck), the pony’s observable reaction at the moment of injection was scored on a 4-point scale similar to that used to score reactions of dogs and cats to intravenous catheter placement [6,8]. A score of 0 indicated no observable reaction; 1 indicated slight skin or muscle flinch without any other head, neck, limb or body movement; 2 indicated mild-to-moderate head, neck, body and/or limb movement; and 3 indicated greater animated movement of any level. 

### 2.5. Statistical Analysis

For each observer, an overall reaction score for each pony was calculated as the mean of their reaction scores for the left and right injections. As an indicator of inter-observer reliability, Fleiss’ kappa procedures were used to evaluate agreement of the two handlers’ independent overall reaction scores. Based on a result within the range considered substantial agreement (Fleiss’ kappa, 0.66), one observer’s data (SM) were randomly selected to complete the statistical evaluation. Significance of differences in reaction score among treatment groups was evaluated using Kruskal–Wallis one-way ANOVA with Mann–Whitney U follow-up all pair-wise comparison procedures. A probability level of <0.05 was considered statistically significant. Data were analyzed using Statistix 10 © (Analytical Software, Tallahassee, FL, USA). Statistical analyses were completed blindly to the identity of the coded treatment groups until it was necessary to perform hypothesized directional follow-up group comparisons.

For further analysis, reaction scores were collapsed into two categories, *no or slight flinch* (overall score of 1 or less) versus *mild, moderate or greater movement* (overall score greater than 1). Chi-square analysis was used to evaluate the significance of difference among the three treatment groups in the proportion of subjects with overall reaction scores greater than 1. The rationale for this two-level categorization is the practical significance of the distinction in terms of efficiently and safely completing the vaccinations, as well as risk of developing problematic aversion to injections. Under typical on-farm conditions for vaccination, horses are hand-held with a halter and lead rope rather than confined in an examination stall. Movement greater than a slight flinch may interrupt the procedure, quickly leading to learned avoidance. Movement may also increase discomfort of the procedure by tearing tissue or requiring repeated piercing of the skin. Importantly, handler response to the horse’s movement and/or interruption of the procedure often includes verbal and physical punishment and in some cases more severe restraint. These additional physical and psychological discomforts associated with injections increase the risk of a negative experience sufficient to lead to problematic learned avoidance behavior, affecting future comfort and compliance with health care procedures. 

## 3. Results

**Figure 2:**
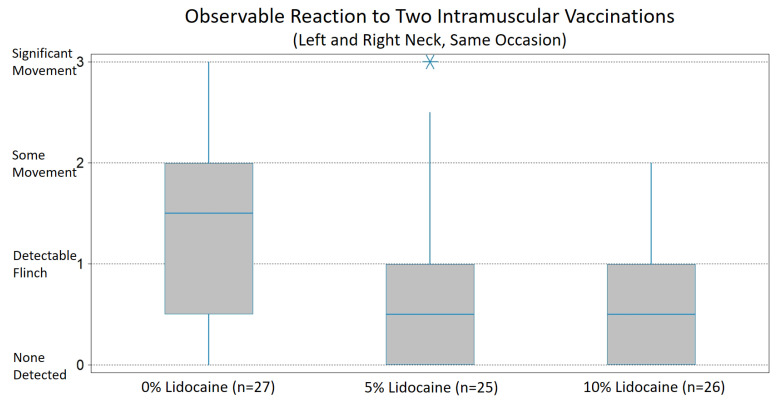
Box spans middle half of data, bisect line = median, whiskers span typical data, * = outlier.

Reaction scores are graphically summarized in Figure 2. Mean reaction scores were 0.84 (se 0.18) for 5% lidocaine solution and 0.62 (se 0.14) for 10% lidocaine solution, compared to 1.30 (se 0.19) for 0% control solution. Reaction scores for both the 5% and 10% lidocaine groups were significantly lower than for the control group (Kruskal–Wallis, *p* < 0.01, Mann–Whitney U, one-tailed, post-comparisons, *p* < 0.05 and *p* < 0.001, respectively). The difference between the 5% and 10% lidocaine groups was not significant. Considering the proportion of subjects with an overall reaction score greater than 1, there were 2 of 25 in the 5% lidocaine group, 5 of 26 in the 10% lidocaine group and 15 of 27 in the 0% lidocaine control group. For both the 5% and 10% lidocaine groups, the difference was significant from the control (chi-squared, *p* < 0.001).

## 4. Discussion

Blind evaluation of two commercially available topical anesthetic products, one with 5% lidocaine and another with 10%, indicates that application of either anesthetic solution to injection sites two minutes in advance of intramuscular vaccination can effectively reduce the behavioral discomfort reaction of horses. The mean reaction scores for ponies to which either a 5% or 10% topical lidocaine solution was applied were significantly lower compared to control ponies. Of practical importance, for both the 5% and 10% lidocaine solutions, the proportion of ponies with a reaction that could be expected to adversely affect efficiency and safety, as well as increase the risk of a horse developing a problematic aversion to injections, was significantly lower compared to the control. In both analyses, the difference between the 5% and 10% lidocaine solutions evaluated was not significant. 

For the ponies used in this study, the authors’ impression was that reactivity to intramuscular injection is generally lower than that of horses in the typical equine practice population. We expect several factors contribute to this perceived difference. As was performed for this study, our routine practice and recommendation for least-stress health care for horses is to use 22 G needles whenever possible for administering intramuscular or intravenous injection of aqueous preparations [11,12]. In equine practice, it is more typical to use 20 G or 21 G or even 18 G needles. In fact, most single-dose equine vaccines marketed in North America are packaged with 20 G needles. In our experience, the finer 22 G needles appear to be conspicuously less uncomfortable for most horses. Nonetheless, even when using 22 G needles in this study, topical lidocaine added benefit. Also of note, we used 1-inch-long needles. It is quite common for 1.5-inch-long needles to be used with horses and to be provided with single-dose vaccines. Further work to address the relative contribution of topical anesthetic and needle gauge and length may be indicated to inform health care providers of the individual and combined benefits of these relatively simple and inexpensive methods that may improve equine patient comfort and tolerance. 

Another factor likely contributing to lower reactivity of the ponies in this study is their lifelong exposure to non-confrontational handling using primarily positive reinforcement behavior modification based on learning principles. While these methods are becoming more widely appreciated and promoted as best practice for animal welfare during health care, they are not yet widely practiced or understood by the horse-owning public, equine professionals or veterinary care staff. Many handlers still rely primarily on aversive and/or coercive handling strategies with horses, including increased punitive restraint, as well as verbal and physical “discipline” for expression of escape or avoidance responses to uncomfortable health care procedures. For example, veterinarians who indicated in a survey that they understood learning principles relevant to veterinary care of horses, when subsequently questioned on specific case scenarios, performed poorly [1]. Similarly, a recent study reported poor agreement among expert horse professionals on behavioral indicators of stress in horses undergoing veterinary procedures [14]. 

For these semi-feral animals, we have found that the holding stall with “wiggle room” safely allows some reactive movement but limits escape. This is different from the typical situation for vaccination of domestically managed horses, where the horse is usually hand-held using a halter and lead in a larger space. This arrangement generally includes greater risk of injury to personnel, interruption of the interaction and even escape from the handler. Any escape or avoidance behavior that interrupts the procedure is, in essence, reinforced by the release of pressure (negative reinforcement). Reduced discomfort of injections may be of even greater benefit under those conditions. To address this question, further work with horses under typical domestic handling experience is underway in our lab.

This study investigated lidocaine solutions with the primary goal of anesthetizing the skin, as piercing of the skin often appears to be the most uncomfortable aspect of intramuscular injection for horses. There are multiple barriers that effectively inhibit absorption through intact skin [15,16]. Accordingly, considerable work has addressed approaches to enhance absorption of topical analgesics, including various formulations of active and complementary components [17]. Reported latency and duration of the analgesic effect vary widely among particular approaches and formulations. A eutectic cream preparation containing 2.5% lidocaine with 2.5% prilocaine (EMLA^®^) is the most widely studied in regard to application to intact skin for injection or placement of indwelling catheters [18]. The latency to achieve analgesia of underlying tissues has been reported to range from 30 to 90 min. In this current work with horses, both the 5% and 10% lidocaine solutions applied to intact skin of the neck were effective at reducing behavior reaction within two minutes of application. Each of these products also includes propylene glycol to enhance absorption through the skin; however, it is unlikely that lidocaine reached the deeper muscle tissues within the two minutes. Certainly, further investigation of effectiveness of these and various other preparations may be worthwhile, particularly for jugular catheter placement or for injection of medications that may cause greater discomfort of underlying tissues than the relatively small-volume aqueous vaccines evaluated here.

In this work, the difference between 5% and 10% topical lidocaine solutions both for mean reaction scores as well as for the proportion of subjects having no or only a slight flinch response was not significant. This is currently of practical significance because 5% topical lidocaine preparations are more readily available. In North America and many other parts of the world, topical lidocaine preparations greater than 5% require a prescription. 

Despite the short duration of iatrogenic discomfort caused by vaccination or other injections, the behavioral consequences for horses and their impact on welfare may be long-lasting. Our experience indicates that horses can begin to develop an injection aversion after only one uncomfortable experience. From that point forward, the typical progression for many horses involves increasingly aggressive handling techniques to attempt to restrain or punish increasingly animated avoidance behavior. This experience can quickly lead to conditioned fear that may generalize beyond injections, making those horses dangerously challenging to handle for other health care procedures. If we can reduce the discomfort of injections, we may be able to avoid development of aversions to injections and other health care procedures, thereby improving welfare both at the time of vaccinations and lifelong.

## 5. Conclusions

In conclusion, this work demonstrates that application of topical anesthetic can effectively reduce the discomfort behavior reaction of horses to intramuscular injection. Both the 5% and 10% lidocaine products commercially available for numbing human skin were effective when applied only two minutes before vaccination, making the procedure practical for routine use. Further work should be helpful in optimizing benefit from these and other topical numbing preparations.

## Data Availability

Not applicable.
